# The Impact of Dietary Fiber as a Prebiotic on Inflammation in Children with Obesity

**DOI:** 10.3390/foods11182856

**Published:** 2022-09-15

**Authors:** Chonnikant Visuthranukul, Tanisa Kwanbunbumpen, Yuda Chongpison, Supakarn Chamni, Ekkarit Panichsillaphakit, Jaraspong Uaariyapanichkul, Settachote Maholarnkij, Sirinuch Chomtho

**Affiliations:** 1 Pediatric Nutrition Research Unit, Division of Nutrition, Department of Pediatrics, Faculty of Medicine, Chulalongkorn University, Bangkok 10330, Thailand; 2 Division of Nutrition, Department of Pediatrics, King Chulalongkorn Memorial Hospital, The Thai Red Cross Society, Bangkok 10330, Thailand; 3 The Skin and Allergy Research Unit, Faculty of Medicine, Chulalongkorn University, Bangkok 10330, Thailand; 4 Biostatistics Excellence Center, Research Affairs, Faculty of Medicine, Chulalongkorn University, Bangkok 10330, Thailand; 5 Natural Products and Nanoparticles Research Unit, Department of Pharmacognosy and Pharmaceutical Botany, Faculty of Pharmaceutical Sciences, Chulalongkorn University, Bangkok 10330, Thailand

**Keywords:** obesity, inflammation, inflammatory cytokines, fecal calprotectin, inulin, prebiotics, functional foods

## Abstract

Background: Obesity is associated with dysbiosis, contributing to inflammation and insulin resistance. Inulin might reduce inflammation by manipulating intestinal microbiota. Objective: We aimed to determine the effects of inulin supplementation on inflammation and assess the relationships of inflammatory cytokines with adiposity and insulin resistance in obese Thai children. Design: Obese Thai children ages 7–15 years were randomly assigned to inulin (intervention), maltodextrin (placebo), and dietary fiber advice groups. All participants received monthly follow-up and identical advice on lifestyle modification for six visits. Body composition was evaluated using bioelectrical impedance analysis. IL-1β, IL-6, TNF-α, and fecal calprotectin were analyzed by ELISA technique at baseline and the final visit. Spearman correlation was used to assess the associations between inflammation and other clinical outcome variables. Results: A total of 155 obese children completed the study (mean age: 10.4 ± 2.2 years, 59% male). All groups showed a significant decrease in BMI z-score, fat mass index (FMI), percent body fat, and trunk FMI. A generalized estimating equation (GEE) model showed significantly decreased IL-1β and TNF-α of 34.8% and 25.8%, (*p* < 0.0001) but increased IL-6 (21.5%, *p* = 0.006) in all groups. There were no significant differences in inflammatory cytokines and fecal calprotectin between groups. Mean IL-6 was higher in obese children with acanthosis nigricans (*p* = 0.048). Only IL-6 was positively correlated with body fat percentage and FMI (r = 0.29, *p* = 0.008 and r = 0.25, *p* = 0.049, respectively). Conclusions: Intensive behavioral modification and frequent follow-up visits were effective methods to reduce BMI and adiposity leading to decreased inflammatory cytokines. The additional benefits of inulin on inflammation could not be demonstrated due to the Hawthorne effect. Among the three cytokines, IL-6 was the most likely mediator relating FM and insulin resistance at baseline; therefore, it could be used as a surrogate marker of inflammation in obese children who are at risk for insulin resistance and metabolic syndrome.

## 1. Introduction

Obesity is a major concern worldwide for all age groups. The prevalence of overweight and obese children and adolescents has risen dramatically from 4% in 1975 to over 18% in 2016 [1]. Moreover, overweight and obesity affect nearly one in three children (29% of boys and 27% of girls) in the WHO European Region based on the data from WHO European Regional Obesity Report 2022 [2]. It is well established that obesity is associated with insulin resistance and metabolic syndrome involving a multifactorial etiology. The gut microbiota may be one of the key environmental factors driving metabolic diseases. Studies have found that obese individuals had lower Bacteroidetes and more Firmicutes than lean subjects [3,4,5].

A high-fat diet and obesity could modulate microbiota and induce alteration of the gut barrier associated with increased absorption and circulation of lipopolysaccharides (LPS), which is an endotoxin from the outer cell membrane of Gram-negative bacteria [6,7]. LPS binds the plasma LPS-binding proteins, activating toll-like receptor 4 (TLR4) on the surface of adipose tissue macrophages. This triggers the expression of genes encoding nuclear factor-κB (NF-κB) and activator protein 1 (AP-1), producing IL-6 and TNF-α. LPS also participates in the inflammasome pathway, activating IL-1β [8]. These mechanisms result in a chronic, low-grade inflammation, which can lead to insulin resistance and metabolic syndrome. One manifestation is acanthosis nigricans, which is caused by increased insulin and insulin-like growth factor leading to keratinocyte and dermal fibroblast proliferation. The three cytokines affect insulin sensitivity, by altering the expression of genes encoding insulin receptor substrate-1 (IRS-1), glucose transporter-4 (GLUT-4), and peroxisome-proliferator activated receptor-α (PPAR-α) [6,9]. Verdam et al. showed that, compared to normal adults, obese individuals had reduced bacterial diversity and a reduced Bacteroidetes/Firmicutes ratio, which was correlated with higher plasma C-reactive protein (CRP) [10].

Previous research has shown that inulin-type fructans (ITFs) have beneficial effects for the treatment of metabolic endotoxemia or low-grade inflammation in obese subjects [11]. Children who consumed ITFs for 16 weeks had a significant decrease in body weight z-score, percent body fat, percent trunk fat, IL-6, and Bacteroides. It also showed a significant increase in *Bifidobacterium* spp. compared to controls [12]. Previous studies in overweight and obese diabetic adults demonstrated that patients receiving ITF for 8 weeks had a significant reduction in body weight, BMI, IL-6, TNF-α, and total fat [13,14]. Moreover, fecal calprotectin, which is a protein secreted from neutrophils and a marker determining intestinal inflammation, increased in obese adults [15]. Fecal calprotectin can normally be found in stool approximately six times higher than in blood circulation. Fecal calprotectin > 50 µg/g indicates local inflammation in the intestine and this value can be used in children aged > 4 years [16]. There is limited evidence on the effects of prebiotics on systemic and local inflammations in obese children. The effects of dietary fiber on inflammation are inconsistent and might differ between ethnicity due to different dietary intake and age groups. Therefore, our objectives were to determine the effect of inulin supplementation on inflammation and assess the relationships of inflammatory cytokines with BMI z-score, adiposity, and insulin resistance in obese Thai children.

## 2. Materials and Methods

### 2.1. Subjects

This study was a randomized double-blinded placebo-controlled trial conducted from August 2017 to July 2020 at the King Chulalongkorn Memorial Hospital (KCMH), Thailand.

The Institutional Review Board of the Faculty of Medicine, Chulalongkorn University approved the study protocol. Participants and their guardians signed informed consent forms prior to study enrollment. This trial was registered at clinicaltrials.gov (NCT03968003) and reported according to the CONSORT statement. Obese Thai children ages 7 to 15 years with a BMI more than 2 standard deviations (SDs) above median as per the WHO growth reference [17] were recruited from the Pediatric Nutrition and the Pediatric Obesity clinics from KCMH and from social media (Chula Kids Club). Exclusion criteria were: syndromic obesity, endocrine causes of obesity (e.g., hypothyroidism, growth hormone deficiency), use of drugs that influenced appetite or body weight (e.g., corticosteroids), and attending other concurrent weight reduction programs.

### 2.2. Study Design

The detailed study design has been published elsewhere [18]. In brief, 165 participants were randomly assigned to 3 groups: placebo group, inulin group, and dietary fiber advice group. For the inulin group, 13 g of isocaloric oligofructose enriched inulin extracted from the Thai Jerusalem artichoke was prescribed daily at approximately 30 min prior to dinner. The placebo group received 11 g of isocaloric maltodextrin, which is a type of carbohydrate without prebiotic properties. The other group was the dietary fiber advice group, which received the specific instruction to consume an appropriate amount of dietary fiber by age [19,20]. All groups received the same advice about a low energy and a low fat diet, engaging in non-weight-bearing exercise, and reducing screen time. All received instructions about behavioral modification. All participants were followed-up monthly for 6 months. The details and flow diagram of the study are reported elsewhere [18].

### 2.3. Dietary Assessment

Dietary intake was assessed by a dietician, using 3-day dietary records. The daily calories, percentage of energy distribution, and nutrient intake were calculated by using the Institute of Nutrition, Mahidol University Calculation-Nutrients (INMUCALs) database version 3.0 (Nakhon Pathom, Thailand) [21].

### 2.4. Anthropometry and Body Composition

Trained personnel performed the anthropometric measurements. Weight, height, waist, and hip circumferences were measured. BMI was calculated as weight in kilograms divided by the square of height in meters (kg/m^2^). BMI z-score was calculated based on WHO 2007 growth reference using WHO Anthroplus program [22]. Body composition was measured by bioelectrical impedance analysis (BIA) using the InBody 770 (InBody Co., Ltd., Chungcheongnam-do, Korea). Fat mass index (FMI) and fat-free mass index (FFMI) were calculated in the same manner as BMI [23].

### 2.5. Inflammatory Cytokines

Venous blood was obtained after a 12-h fast to evaluate biochemical parameters at the first and sixth visits of the study. Plasma samples were stored at −80 °C until analysis. Plasma inflammatory cytokines were quantified using Bio-Plex ProTM Human Cytokine Assays (Bio-Rad, Hercules, CA, USA). The Bio-Plex Suspension Array System, which was based on Luminex xMAP technology, was a multiplex flow cytometric based system that utilized up to 100 color-coded 8 µm magnetic bead sets. Each bead set was internally dyed with different ratios of two spectrally distinct fluorophores. Each of the bead sets could be conjugated with a unique antibody, antigen, enzyme, and substrates. The conjugated beads were pooled together in the wells of a microplate with the sample to be tested, followed by the addition of a detection antibody, forming a capture immunoassay that was read by the Bio-Plex suspension array reader. Each separate reaction was identified and quantified based on the bead color.

### 2.6. Fecal Calprotectin

Fecal samples were collected by the parents at home and transported to the hospital on ice within 1 h. They were stored at −80 °C until analysis. The frozen samples were allowed to thaw slowly and warm up to room temperature before analysis. Fecal concentration of calprotectin was measured by enzyme-linked immunosorbent assay (ELISA) test (IDK^®^ Calprotectin ELISA, Bensheim, Germany) as per protocol. 

### 2.7. Metabolic Profiles

Fasting blood sample was analyzed for plasma glucose (FPG), serum total cholesterol, HDL-C, LDL-C, triglyceride, and alanine aminotransferase (ALT). The details of these analysis are reported elsewhere [18].

### 2.8. Statistical Analysis

The normality of each variable was evaluated using the Shapiro–Wilk test. Baseline characteristics were compared among the three groups using one-way analysis of variance and chi-square tests, whenever it was appropriate. Descriptive statistics for categorical and continuous variables were presented as frequency (%) and mean ± standard deviation (SD), respectively. Within-group and between-group comparisons for anthropometry, metabolic profiles, and other variables were conducted using the paired t-test and one-way ANOVA as well as the generalized estimating equation (GEE) model, respectively.

Three cytokines, IL-1β, IL-6, and TNF-α, and fecal-calprotectin were log-transformed. Geometric means and %CV were used to describe log-transformed cytokine levels and log-transformed fecal calprotectin. To evaluate the change in log-transformed cytokines over time and between-group differences, the GEE model with an interaction term between dietary groups and time was used. Spearman correlation coefficients was used to assess the associations between inflammatory cytokines and body composition variables. The impact of physical activity on fecal calprotectin over the 6-month period were evaluated using the GEE model with an interaction term used between different physical activity status and time point.

All statistical tests were two-sided and *p*-value < 0.05 was considered statistically significant. The data were analyzed using STATA version 17.0 (STATA Statistical Software: Release 17. College Station, TX, USA: STATA Corp LLC. 2021).

## 3. Results

A total of 165 obese Thai children participated in the study and were randomly allocated into placebo, inulin, and dietary fiber advice groups. The flow diagram of the study was reported elsewhere [18]. Ten participants (6%) dropped out of the study. A total of 155 obese Thai children completed the study (mean age: 10.4 ± 2.2 years, 59% male). The demographic data and baseline characteristics of all groups are illustrated in Table 1. There were no significant differences in baseline anthropometry, clinical data, nutrient intake, physical activity, inflammatory cytokines, and fecal calprotectin (*p* > 0.05).

Within group comparison found that BMI-z score significantly decreased after the intervention in all groups (*p* < 0.0001). Caloric and fat intake significantly decreased with a significantly increased dietary fiber intake in all groups after the intervention (*p* < 0.01, except dietary fiber group, *p* = 0.03). There was a significant decrease in FMI, percent body fat, and trunk FMI in all groups (*p* < 0.01). There were no significant changes within groups in FPG, lipid profiles, and ALT after the intervention.

There were no differences in the changes in these clinical outcomes after the intervention among the three groups by ANOVA and GEE model.

### 3.1. Inflammatory Cytokines

**Figure 1:**
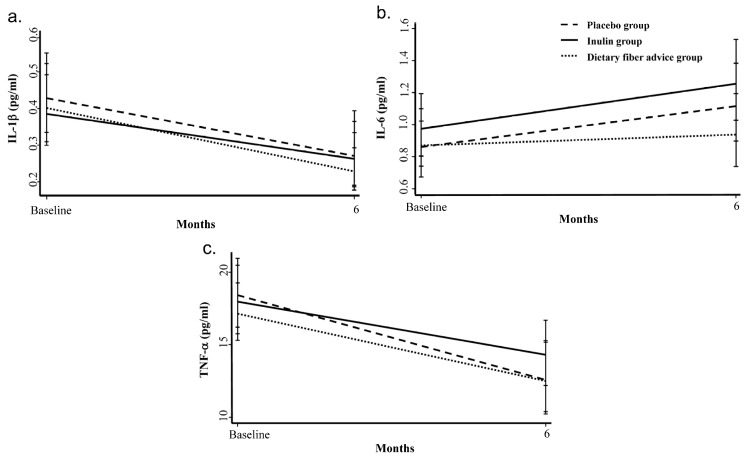
Changes in inflammatory cytokines: (**a**) IL-1β, (**b**) IL-6, and (**c**) TNF-α over the 6-month intervention. IL-1β, IL-6, and TNF-α data were not normally distributed. The log transformation was used to make highly skewed distributions less skewed and then the comparison was made among geometric means (anti-log of the arithmetic mean of log-transformed value). Geometric mean for IL-1β and TNF-α decreased by 34.8% and 25.8%, respectively. Geometric mean for IL-6 increased by 21.5% in all groups. There was no difference between groups by GEE models. GEE, generalized estimating equation; IL, interleukin; TNF, tumor necrosis factor.

All analyses followed the intention-to-treat principle. IL-1β, IL-6, and TNF-α data were not normally distributed. Then, log transformation was used to make highly skewed distributions less skewed and then the comparison was made among geometric means. Baseline IL-1β, IL-6, and TNF-α were not significantly different between groups (Table 1). There were also no significant difference in IL-1β, IL-6, and TNF-α between groups over the 6-month period by the GEE models. Geometric mean for IL-1β and TNF-α decreased by 34.8% and 25.8%, respectively, but IL-6 increased by 21.5% in all groups (Figure 1).

**Figure 2:**
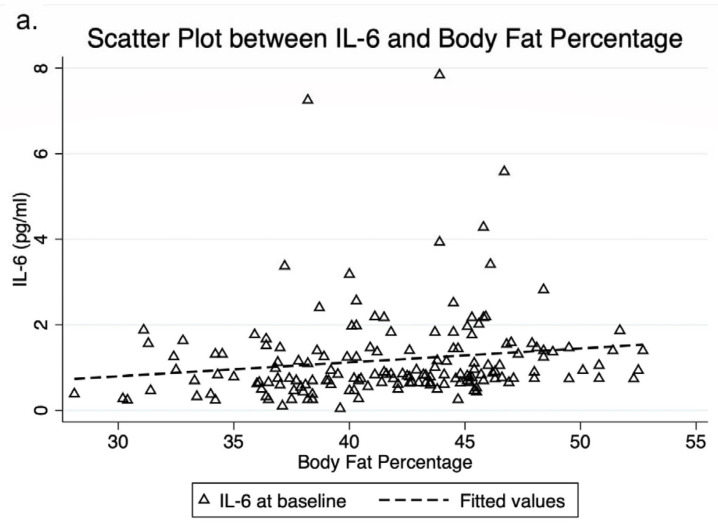

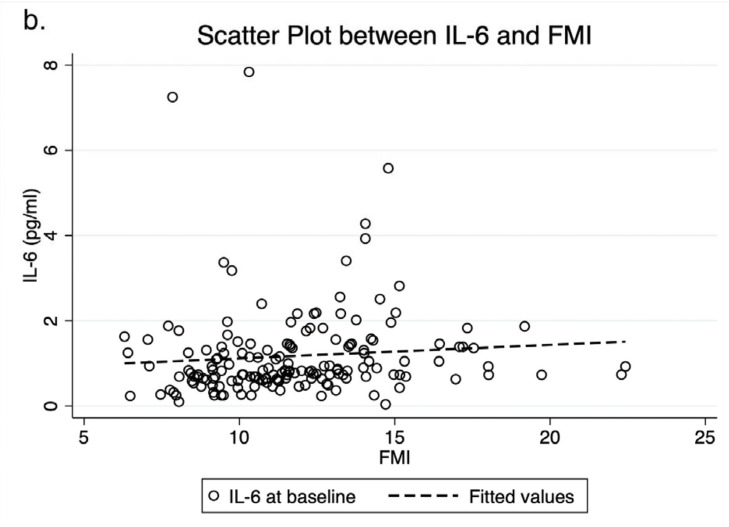
The relationship of IL-6 with body fat percentage (**a**), and FMI (**b**). IL-6 was positively correlated with body fat percentage and FMI (r = 0.29, *p* = 0.008 and r = 0.25, *p* = 0.049, respectively) by Spearman correlation coefficients. FMI, fat mass index; IL, interleukin.

Mean IL-6 was significantly higher in obese children with acanthosis nigricans than those without this condition (mean difference 0.547 pg/mL (*p* = 0.048)). Furthermore, among the three cytokines, only IL-6 was positively correlated with body fat percentage and FMI (r = 0.29, *p* = 0.008 and r = 0.25, *p* = 0.049, respectively) by Spearman correlation coefficients (Figure 2). IL-1β and TNF-α were not associated with acanthosis nigricans, body fat percentage, FMI, FPG, and lipid profiles at baseline.

### 3.2. Fecal Calprotectin

The stool samples for fecal calprotectin were collected from subgroups of 27 obese children in each group (a total of 81 children) at baseline and the final visit. There was no significant difference in the mean difference of fecal calprotectin among the three groups. The change of acanthosis nigricans in 81 obese children was classified as changed and unchanged groups after the 6-month period. No significant difference in the change of fecal calprotectin between both groups was observed (data not shown). Changes in physical activity intensity from baseline to the 6th month in 81 obese children were classified as unchanged, increased (from low to moderate-to-high intensity), and decreased physical activity. There was no significant difference in the change of fecal calprotectin among the groups (data not shown).

## 4. Discussion

To our knowledge, this was the largest randomized controlled trial to assess inflammatory cytokines and fecal calprotectin after prebiotic intervention in obese children. Our study found that BMI z-score, FMI, trunk FMI, and percent body fat significantly decreased after the intervention in all groups, but there were no differences between groups. We demonstrated that IL-1β and TNF-α were significantly decreased in all groups, while IL-6 experienced an increase. There were no differences in the changes of these cytokines among the three groups. Importantly, mean IL-6 was significantly higher in obese children with acanthosis nigricans. Additionally, only IL-6 was positively correlated with body fat percentage and FMI.

Existing research exploring the changes in inflammatory cytokines after a prebiotic intervention in childhood obesity is scarce. Nicolucci et al. [12] did not find a significant decrease in IL-1β and TNF-α in the inulin and placebo groups after the 4-month intervention. The effect size of a 2.4% body fat reduction in their study was smaller than the 3.1–5.5% reduction among groups in our study. We postulated that the reduced body fat might lead to a decrease in inflammatory cytokines, IL-1β and TNF-α, after the intervention period despite no demonstrated between group differences. This indicates that adipose tissue macrophages could be the source of inflammatory cytokines in obese individuals [6,8]. Nicolucci et al. [12] found that the obese children had a significant reduction of IL-6 after prebiotic intervention compared to the placebo (*p* = 0.01), in contrast to our study. We propose that exercise and sedentary activity may impact the changes in IL-6. The increase in IL-6 could have resulted from an increase in physical activity in all groups. There have been some published data supporting that an increase in IL-6 is associated with increased exercise. During exercise, IL-6 was produced by skeletal muscle depending on the mode, frequency, duration, and intensity of exercise [24,25].

Previous studies have shown that several factors, apart from exercise and physical activity, such as a high-fat diet, gut microbiota, prebiotics, and insulin resistance, could affect a change in inflammatory cytokines [6,7,9,26,27,28]. High-fat diets and dysbiosis were associated with obesity [27]. The decreased Bacteroidetes:Firmicutes ratio was found in people with obesity who usually consume high-fat diets [6]. The mechanisms that linked to inflammation are attributed to the scavenger receptor class B type 1 (SR-BI). This receptor binds to lipopolysaccharide (LPS) and then the LPS-binding protein activates the receptor protein CD14, which binds Toll-like receptor 4 (TLR4) at the surface of macrophages [6,7,26]. They trigger NF-κB and AP-1, produce IL-6 and TNF-α that affect insulin sensitivity by altering the expression of genes encoding IRS-1, GLUT-4, and PPAR-α [6,9,28,29,30] and lead to insulin resistance [31]. Based on this hypothesis, inulin intake may help to reduce inflammation by modulating dysbiosis in obese individuals. Dewulf et al. [32] showed that ITFs supplement in obese women increased *Bifidobacterium* and *Faecalibacterium prausnitzii*, which were negatively correlated with serum LPS. Everard et al. [33] demonstrated that ITFs treatment restored the abundance of *Akkermansia muciniphila* which is a mucin-degrading bacteria in the mucous layer. They improved gut barrier leading to a decreased level of endotoxemia. These proposed mechanisms might be a cause of the reduction in the inflammatory cytokines after inulin supplementation. Another possible reason that we could not demonstrate a significant difference in inflammatory cytokines between groups was that the intensive behavior modification and monthly follow up attenuate the differential effects from inulin supplementation.

Baseline inflammatory cytokines might also have affected our results. A previous study in adults suggested that the young and healthy population did not show sufficient baseline cytokines necessary to detect a significant modulation by prebiotic intervention. Lecerf et al. [34] performed a study to determine prebiotic effects on LPS and immune parameters in 60 healthy subjects aged 18–24 years during a 4-week intervention period. They found that LPS was significantly lower in the inulin-xylo-oligosaccharide mixture (INU–XOS) group compared to the placebo group (*p* = 0.026), but there was not a sufficient baseline expression of cytokines to detect significant change after the intervention. LPS-challenged whole blood was collected from subjects to understand the priming of the immune response. Results showed that consumption of INU-XOS reduced IL-1β gene expression with an increased IL-13 gene expression, suggesting a capacity to reduce the intensity of an acute pro-inflammatory reaction. This indicates that obese children in our study may not have had sufficient baseline cytokines to detect a significant difference between groups.

In our study, IL-6 was significantly higher in obese children with acanthosis nigricans. This may be explained by the relationship of inflammatory cytokines with insulin resistance and diabetes. Mirza et al. [35] determined the associations between diabetes and inflammatory cytokines and adipokines in 367 Mexican Americans with type 2 diabetes mellitus (T2DM). They demonstrated that median IL-6 and TNF-α were higher in diabetes compared to non-diabetes (*p* < 0.01), suggesting that diabetes was strongly associated with elevated levels of IL-6 and TNF-α. In addition, Miyazaki et al. [27] evaluated the relationship between TNF-α and peripheral tissue sensitivity to insulin. They showed that TNF-α positively correlated with FPG and fasting plasma insulin in normal glucose tolerance and impaired glucose tolerance subjects. An increase in TNF-α was associated with insulin resistance, increased plasma glucose, and insulin prior to the onset of T2DM. We presumed that IL-6 and TNF-α were associated with a pro-inflammatory state leading to insulin resistance and diabetes. However, we could only demonstrate such an association with IL-6 but not TNF-α.

Based on the associations between inflammatory cytokines and adiposity, our findings highlighted the relationships of IL-6 with body fat percentage and FMI. A previous study found that production of inflammatory cytokines in visceral adipose tissues increased insulin resistance [6]. Increased fat accumulation and lipotoxicity stimulate cytokine production that is primarily implicated in innate immunity [36,37]. This process leads to a chronic, low-grade inflammatory status, a mature immune cell activation, especially in adipose tissues, and also an induction of adipocyte recruitment and activation, promoting the inflammatory process [38,39]. This suggests that there is a relationship between acanthosis nigricans, a sign of insulin resistance, adiposity, and the increase in IL-6. We postulated that IL-6 is a central player in the regulation of inflammation. IL-6 releases from adipose tissue, resulting in a subclinical increase in plasma levels. Among the three inflammatory cytokines in our study, IL-6 might be the most closely related mediator to FM. Therefore, IL-6 at baseline could be used as a surrogate marker of inflammation in obese children.

Fecal calprotectin is an indicator of local inflammation in the intestine. Hester et al. [40] determined the effect of anthocyanin and prebiotic blend on intestinal inflammation in 41 obese participants aged 18–50 years who agreed to maintain regular diet and exercise for 8 weeks. They did not find a significant decrease in fecal calprotectin after the intervention, similar to our study. The possible reason was that the sample size in our study might be too small to detect the difference because there were several factors that could affect the change of fecal calprotectin, such as intestinal microbiota composition that is associated with both local and systemic inflammations in obesity. A longer study duration may also have resulted in a significant decrease in fecal calprotectin because the changes in microbiota composition and diversity possibly have a larger impact on the progression of low-grade inflammation [10]. Physical activity might be one of the factors affecting fecal calprotectin. A previous study showed that physical activity protected against onset of inflammatory bowel disease [41]. Further research on the effect of physical activity on fecal calprotectin in obesity is warranted.

This appears to be the largest and the first study documenting the change in both systemic and local inflammations after inulin supplementation in obese children. We demonstrated a significant relationship of IL-6 with FM and insulin resistance at baseline. These results support the use of IL-6 as a marker of systemic inflammation in obese children. Another strength of our study was the use of a double-blinded design which helped minimize bias. We proposed several reasons why we were not able to demonstrate a differential effect of inulin on the changes in inflammatory cytokines. Firstly, the increased exercise of the groups may have confounded the changes in IL-6 and this should be interpreted with caution. Secondly, intensive behavioral modification may significantly reduce BMI and adiposity in all groups and might attenuate the effect of inulin supplementation. Thirdly, most of our obese children may not have had sufficient baseline inflammatory cytokines to show a reduction after the intervention.

## 5. Conclusions

Intensive behavioral modification and regular follow-up are effective methods to reduce BMI and adiposity in all groups, which may lead to decreased inflammatory cytokines. Despite showing a subtle effect on inflammation, our findings highlight the associations between acanthosis nigricans, adiposity, and IL-6. Among the three inflammatory cytokines, IL-6 might be the most likely mediator relating FM and insulin resistance at baseline. Therefore, IL-6 could be used as a surrogate marker of inflammation in obese children who are at risk for insulin resistance and metabolic syndrome. Further studies are needed to understand the direct effect of physical activity, the mechanism of gut microbiota, and insulin resistance on systemic and local inflammations in obese children. Further study in gene expression might help to explain changes in inflammation in this population.

## Figures and Tables

**Table 1:** Baseline characteristics of obese children at the first visit (*n* = 165).

	Placebo Group * (*n* = 55)	Inulin Group * (*n* = 55)	Dietary Fiber Advice Group * (*n* = 55)
Age, years	10.7 ± 2.4	10.3 ± 2.1	10.4 ± 2.0
Sex, male, %	56.4	54.6	67.3
BMI, kg/m^2^	28.5 ± 4.6	28.3 ± 4.5	27.4 ± 3.4
BMI for age z-score	3.2 ± 1.1	3.3 ± 1.0	3.2 ± 0.95
Waist circumference, cm	90.6 ± 10.8	89.9 ± 11.3	88.6 ± 10.0
SBP, mmHg	115.3 ± 10.0	114.1 ± 8.3	117.4 ± 11.1
Acanthosis nigricans, %	83.6	76.4	80.0
Tanner stage			
Stage 1, %	56.36	65.45	69.09
Stage 2, %	10.91	20	12.73
Stage 3, %	20	12.73	10.91
Stage 4, %	10.91	1.82	7.27
Stage 5, %	1.82	0	0
BIA			
FMI, kg/m^2^	12.0 ± 2.9	11.9 ± 3.1	11.4 ± 2.7
FFMI, kg/m^2^	16.3 ± 2.5	16.2 ± 1.9	16.0 ± 1.7
Percent body fat, %	42.0 ± 5.5	41.9 ± 4.8	41.3 ± 4.9
Trunk FMI, kg/m^2^	5.7 ± 1.4	5.7 ± 1.5	5.5 ± 1.3
VFA, cm^2^	133.9 ± 39.3	128.8 ± 42.0	125.3 ± 39.7
Total nutrient intake			
Caloric intake, kcal/day	1419 ± 537	1470 ± 571	1463 ± 513
Protein intake, g/kg/day	1.53 ± 0.70	1.54 ± 0.57	1.67 ± 0.66
Dietary fiber, g/1000 kcal	2.8 ± 1.9	2.9 ± 2.3	2.6 ± 1.9
Fat intake, g/day	56.9 ± 28.0	60.9 ± 31.1	58.3 ± 27.4
Cholesterol intake, mg/day	300 ± 198	316 ± 250	330 ± 236
Caloric distribution (%C:P:F)	48:16:36	48:16:36	47:17:36
Metabolic profiles			
Total cholesterol, mg/dL	189.8 ± 29.0	189.6 ± 33.7	189.3 ± 32.8
LDL-C, mg/dL	129.5 ± 27.2	130.4 ± 36.7	126.9 ± 30.5
HDL-C, mg/dL	50.1 ± 9.2	50.4 ± 10.2	53.2 ± 9.1
Triglyceride, mg/dL	103.8 ± 52.6	99.3 ± 36.5	101.6 ± 53.2
ALT, U/L	32.8 ± 32.5	31.3 ± 22.8	27.1 ± 17.2
FPG, mg/dL	82.6 ± 5.9	83.7 ± 5.5	83.2 ± 7.4
Inflammatory cytokines ^†^			
IL-1β, pg/mL	0.33 (110.6)	0.31 (108.2)	0.29 (118.8)
IL-6, pg/mL	0.99 (108.2)	1.10 (80.0)	0.89 (63.0)
TNF-α, pg/mL	15.20 (48.8)	15.70 (49.2)	14.40 (43.0)
Fecal calprotectin, µg/g ^†^	81.00 (124.26)	93.55 (104.88)	76.78 (125.08)

These data showed means ± SD or %. ^†^ Geometric mean (%CV) * One-way ANOVA was used to evaluate continuous variables and Chi-square was used to evaluate categorical variables. ALT, alanine aminotransferase; BIA, bioelectrical impedance analysis; C, cholesterol; FPG, fasting plasma glucose; FFMI, fat-free mass index = fat-free mass (kg)/height (m^2^); FMI, fat mass index = fat mass (kg)/height (m^2^); IL, interleukin; SBP, systolic blood pressure; TNF, tumor necrosis factor; VFA, visceral fat area.

## Data Availability

The data presented in this study are available on request from the corresponding author.

## Abbreviations

ALT, alanine aminotransferase; BIA, bioelectrical impedance analysis; BMI, body mass index; CRP, C-reactive protein; FPG, fasting plasma glucose; FMI, fat mass index; FFMI, fat-free mass index; GEE, generalized estimating equation; HDL-C, high density lipoprotein cholesterol; IL-1β, interleukin-1β; IL-6, interleukin-6; LDL-C, low density lipoprotein cholesterol; LPS, lipopolysaccharides; SBP, systolic blood pressure; SDs, standard deviations; TLR4, toll-like receptor 4; TNF-α, tumor necrosis factor-α; T2DM, type 2 diabetes mellitus; VFA, visceral fat area; XOS, xylo-oligosaccharide.
